# Lessons From Disruption: A New Normal for Families and Care Partners

**DOI:** 10.1093/geroni/igab046.1126

**Published:** 2021-12-17

**Authors:** Beth Fields

**Affiliations:** University of Wisconsin-Madison, Madison, Wisconsin, United States

## Abstract

COVID-19 has forced community-dwelling older adults to rely on family members and care partners more than ever before for support. Often at the expense of their own health and well-being, family members and care partners help older adults manage physical and psychosocial needs, navigate a complex, ever-changing healthcare system, and follow public health guidelines. Given the increasing demands and poor outcomes, there is no better time than now to develop policies and practices that better recognize and support family members and care partners of older adults. To inform policy and practice development, this symposium will present findings from a literature review of peer-reviewed studies published from 2019 through 2021 that identifies and addresses challenges and opportunities related to caregiving for an older adult in a pandemic. The experiences of the past year demonstrate that the new normal needs to recognize and support family members and care partners.

